# Immunological characteristics of MAV/06 strain of varicella-zoster virus vaccine in an animal model

**DOI:** 10.1186/s12865-022-00503-6

**Published:** 2022-06-03

**Authors:** Duckhyang Shin, Younchul Shin, Eunmi Kim, Hyojung Nam, Haiyan Nan, Jaewoo Lee

**Affiliations:** 1 GC Biopharma Corp., 107, Ihyeon-ro 30beon-gil, Giheung-gu, Yongin-si, Gyeonggi-do Republic of Korea; 2 MOGAM Institute for Biomedical Research, 107, Ihyeon-ro 30beon-gil, Giheung-gu, Yongin-si, Gyeonggi-do Republic of Korea; 3 Graduate School of Pharmaceutical Sciences, Ewha Womans University, 52, Ewhayeodae-gil, Seodaemun-gu, Seoul, 03760 Republic of Korea

**Keywords:** Varicella zoster virus, MAV/06 vaccine, Serological cross-reactivity, Cellular immune response

## Abstract

**Background:**

Varicella-zoster virus (VZV) is a pathogen that causes chickenpox and shingles in humans. Different types of the varicella vaccines derived from the Oka and MAV/06 strains are commercially available worldwide. Although the MAV/06 vaccine was introduced in 1990s, little was known about immunological characteristics.

**Results:**

Here, we evaluated B and T cell immune response in animals inoculated with the Oka and MAV/06 vaccines as well as a new formulation of the MAV/06 vaccine. A variety of test methods were applied to evaluate T and B cell immune response. Plaque reduction neutralization test (PRNT) and fluorescent antibody to membrane antigen (FAMA) assay were conducted to measure the MAV/06 vaccine-induced antibody activity against various VZVs. Glycoprotein enzyme-linked immunosorbent assay (gpELISA) was used to compare the degree of the antibody responses induced by the two available commercial VZV vaccines and the MAV/06 vaccine. Interferon-gamma enzyme-linked immunosorbent spot (IFN-γ ELISpot) assays and cytokine bead array (CBA) assays were conducted to investigate T cell immune responses. Antibodies induced by MAV/06 vaccination showed immunogenicity against a variety of varicella-zoster virus and cross-reactivity among the virus clades.

**Conclusions:**

It is indicating the similarity of the antibody responses induced by commercial varicella vaccines and the MAV/06 vaccine. Moreover, VZV-specific T cell immune response from MAV/06 vaccination was increased via Th1 cell response. MAV/06 varicella vaccine induced both humoral and cellular immune response via Th1 cell mediated response.

**Supplementary Information:**

The online version contains supplementary material available at 10.1186/s12865-022-00503-6.

## Background

Varicella-zoster virus (VZV) is one of the most common pathogens that affects humans [1, 2]. The virus causes chickenpox with its initial infection and herpes zoster (shingles, or simply zoster) after later reactivation in the body, induced by waning VZV-specific T cell response [1, 2]. Live-attenuated VZV vaccines have been developed and have been used for decades to prevent chickenpox [3–6]. A zoster vaccine, a highly concentrated form of VZV vaccine [7–9], has recently become available [10].

T cell-mediated immunity is involved in protection of chickenpox as well as zoster [11]. In immune compromising conditions such as aging, the reduction of VZV-specific immune memory CD4+ T cells has been observed. The impaired immunity to VZV can lead to the reactivation of the initial infectious virus, which can be followed by a zoster outbreak [12–14]. In addition, T cell immunity is also crucial in primary VZV infection [15]. Children suffering from immune deficiencies with cellular immunity can be more severe complications by varicella infection, not likely with humoral immunity such as agammaglobulinemia [16–19].

There are 5 major clades and two provisional clades (VI and VII) of VZV that have been identified [20, 21]. Several studies have demonstrated a distinctive geographic distribution of the 5 major VZV genotypes [22, 23]: Clades 1 and 3 are common in Europe and North America; clade 2 has been found in Asia; clade 5 is common in India and Africa; and clade 4 is present in Europe and other areas. The Oka strain, the vaccine strain used in live-attenuated VZV vaccine and zoster vaccine, was isolated in Japan and belong to clade 2 along with most other virus isolates from Japanese and Korean [24–26].

Another VZV vaccine strain, designated as MAV/06, was developed by attenuation of a wild-type isolate obtained from a Korean patient suffered with chickenpox in Seoul [3]. MAV/06 vaccine (Suduvax® as its trade name) has been commercialized in Korea since 1994 and globally since 1998. MAV/06 strain is genetically similar to Oka strain and is also clustered as clade 2 [25]. Although the MAV/06 strain has been used to produce VZV vaccines for more than 20 years, few studies have compared the characteristics of the immunological responses among different VZV strains.

A new MAV/06-based vaccine, BARYCELA®, has been developed and was approved in early 2020 by the Ministry of Food and Drug Safety in Korea. We evaluated the cross-reactivity of antibodies induced by the MAV/06 virus with VZV isolates of various genotypes. In addition, we compared both the humoral and cellular immunogenicity generated by MAV/06 vaccine to those of other VZV vaccines, including those derived from the Oka and MAV/06 viral strains.

## Methods

### Viruses and cells

MRC-5 cells were purchased from ECACC (European Collection of Authenticated Cell Cultures) and maintained in Dulbecco’s modified Eagle’s medium (DMEM; Invitrogen Life Technologies) supplemented with 10% fetal bovine serum (FBS; Gibco) and sodium pyruvate. VZV YC strains and Jena strains were kindly provided from Dr. Hosun Park (Youngnam University, Korea) and Dr. Andreas Sauerbrei (Jena University, Germany), respectively. The 8 VZV YC isolates were clade 2 genotypes [27] and the 6 Jena VZV isolates were clustered into the major VZV clades 1, 3 and 5 [21] (Table 1). VZV viruses were propagated in MRC-5 cells, and titer for infectious viruses were determined with plaque assay on MRC-5 cells.

**Table 1:** Virus isolates used in this study

Strain name	Strain origin	Clade	Sampling date	Patient information (Sex, Age)	Accession No	References
YC01	Zoster	2	19 June 2012	M, 40	KJ767491.1	Kim et al. [27]
YC02	Zoster	2	18 July 2012	M, 3	KJ767492.1	Kim et al. [27]
YC03	Zoster	2	07 Aug. 2012	F, 8	KJ808816.1	Kim et al. [27]
YC04	Zoster	2	02 May 2013	F, 76	–	Kim et al. [27]
YC05	Zoster	2	01 July 2013	F, 52	–	Kim et al. [27]
YC06	Zoster	2	09 July 2013	M, 73	–	Kim et al. [27]
YC07	Zoster	2	22 July 2013	M, 80	–	Kim et al. [27]
YC08	Zoster	2	24 July 2013	M, 66	–	Kim et al. [27]
Jena 4 (432/2008)	Zoster	1	07 Feb.2008	M, 57	JN704695.1	Sauerbrei et al. [31]
Jena 6 (1883/2007)	Varicella	1	07 Nov.2007	F, 4	JN704694.1	Sauerbrei et al. [31]
Jena 12 (2308/2003)	Varicella	3	22 Nov.2003	F, 5	JN704699.1	Sauerbrei et al. [31]
Jena 16 (52/2007)	Zoster	3	08 Jan.2007	M, 18	JN704701.1	Sauerbrei et al. [31]
Jena 26 (446/2007)	Varicella	5	15 Mar.2007	M, 1	JN704707.1	Sauerbrei et al. [31]
MAV/06	Varicella	2	1989	M, 3	JF306641.2	Lee et al. (2011)

To demonstrate the cross-reactivity, various strains of wild type VZV were used along with an attenuated type VZV, MAV/06

### Animal experiment

Male Hartley guinea pigs weighing 200–250 g were purchased from Japan SLC (Japan). Thirty guinea pigs were injected subcutaneously in the scruff of the neck with the MAV/06 vaccine containing approximately 45,000 plaque forming units (PFU) 2 times with a 3-week interval between injections. Blood was collected by cardiac puncture three weeks after second immunization. Sera were pooled and stored at − 70 °C until tested for cross-reactivity.

Five to 6-week-old female C57BL/6 mice were purchased from Orient Bio (Korea). 4 heads of C57BL/6 female mice were used for the mouse experiment. MAV/06 vaccines were prepared with low (~ 600 PFU/0.1 mL), medium (~ 2000 PFU/0.1 mL), and high (~ 4000 PFU/0.1 mL) viral titers. Commercialized vaccines were used as positive control: Suduvax® (Vx1) at a minimum of 1400 PFU/0.5 mL and Zostavax® (Vx2) at a minimum of 19,400 PFU/0.5 mL. Animals were immunized intramuscularly with 0.1 mL of vaccine formulations in thigh muscle of the hind limb 2 times with a 3-week interval between injections after random allocation. Animals were sacrificed two weeks after the second immunization. The sera and spleen were collected from the sacrificed animals.

The sample size was determined by previous experiments, and in previous experiments it was determined that this sample would be appropriate. All animals were anesthetized with isoflurane using closed chambers. Animals were monitored every day and 20% of weight loss was considered for humane endpoints. According to the internal guideline, 20% of weight loss was considered for the criteria. During this study, there were no cases of animals that died.

All experiments including the procedures used and the care of animals were approved by the Institutional Animal Care and Use Committee in GC Biopharma Corp (approval No. 2018002). In addition, we confirm that all methods were carried out in accordance with the Laboratory Animals Welfare Act, the Guide for the Care and Use of Laboratory Animals and the Guidelines and Policies for Rodent Experiments provided by the IACUC (Institutional Animal Care and Use Committee), and all methods are reported in accordance with ARRIVE guidelines for the reporting of animal experiments.

### Plaque reduction neutralization test

Plaque reduction neutralization tests (PRNT_50_) were performed as previously described [28]. Briefly, twofold dilutions of heat-inactivated guinea pig sera, from fourfold through 128-fold, were each mixed with an equal volume of diluted VZV isolates at 100 PFU/0.1 mL. The mixtures were incubated for 1 h at 37 °C. Two hundred microliters of the mixture were added to 6 × 10^5^ MRC-5 cells seeded in wells of 6-well culture plates. The plates were incubated for 60 min at 37 °C with agitation. DMEM with 2% FBS were overlaid and incubated for 5 days. After removing the overlays, a 0.5% crystal violet solution in 25% methanol was added to the cells. The number of stained plaques were counted. PRNT_50_ titers were determined as the reciprocal of the serum dilution that demonstrated a 50% reduction in plaque counts.

### Fluorescent antibody to membrane antigen (FAMA) test

FAMA assay was performed with slight modification from previously described methods [29]. Briefly, MRC-5 cells were grown to confluency in 175 T flasks and infected with VZV isolates at 0.003 m.o.i. When cytopathic effects were observed in ~ 50- 60% of the cells, the cells were washed three times with PBS. Infected cells were detached from the flasks and incubated with serially-diluted sera at room temperature for 30 min. After washing with PBS, cell preparations were incubated with Alexa Fluor^®^488 goat anti-guinea pig IgG (Invitrogen) and mounted on slides. After overlaying with mounting media containing DAPI, the slide was covered with a cover-glass and observed under a fluorescence microscope (Nikon). Titers were defined as the reciprocal of the highest dilution causing bright fluorescent ring around the surface of cells. Titers of ≥ 1:4 were considered positive. Pooled sera from MAV/06 immunized guinea pigs were pre-treated with MRC-5 cells before incubation with virus-infected cells in order to remove immune responses to cellular debris within vaccine ingredients. Non-specific reactions to mock-infected cells were confirmed using both vaccine groups.

### Glycoprotein enzyme-linked immunosorbent assay (gpELISA)

Microplate (Corning) were coated with 1 μg/mL of VZV glycoprotein (QED BIO) in PBS at 4 °C overnight. Plates were washed with wash buffer (0.05% Tween in PBS) and the wells blocked with ELISA assay buffer (1% BSA, 0.1% Tween in PBS) for 2 h. A thousand-fold dilution of sera from immunized mice were added to the wells and incubated for 2 h. Plates were washed and incubated with HRP-conjugated secondary antibody mouse IgG (Southern Biotech) for 1 h. After the final wash, TMB substrate (KPL) was added to each well and incubated for 15 min. The reactions were stopped with TMB stop solution (KPL) and the 450 nm absorbance determined using a spectrophotometer (Molecular Devices, USA) and data were analyzed using SoftMaxPro (Molecular Devices, USA).

### Interferon-gamma enzyme-linked immunosorbent spot (IFN-γ ELISpot) assay

Spleen cells were isolated from the immunized mice and suspended at a final concentration of 5 × 10^6^ per mL in RPMI 1640 medium (Gibco) supplemented with 10% (vol/vol) heat-inactivated FBS (Gibco), antibiotic–antimycotic solution (anti-anti; Gibco), 2 mM Glutamax (Gibco), 1 mM sodium pyruvate (Gibco), and 55 μM 2-mercaptoethanol (Gibco). Multiscreen-HA filter plates were coated with anti-mouse IFN- γ capture antibody (R&D systems) at 4 °C overnight and blocked with RPMI 1640 medium with 10% FBS. Cells were added with intact virions (VZV lysate; Mycrobix), recombinant glycoprotein E (gE), glycoprotein I (gI) (gE and gI from Peptron), IE63 (Genscript), and overlapped peptide (IE63 OLP from JPT), and incubated overnight at 37 °C. The plates were washed with PBS (3x) and biotinylated anti-mouse IFN- γ detection antibody (R&D systems) was added. After washing, streptavidin was conjugated and 3-amino-9-ethylcarbazole (AEC; BD) were added to the plates. The reaction was stopped by rinsing with tap water. Spot-forming units (SFU) were read using ELISPOT reader (Autoimmune Diagnnostika). Adjusted SFU were obtained by subtraction of mock-stimulated counts (mock lysate for VZV lysates, medium for recombinant protein, and DMSO for OLP).

### Cytokine bead array (CBA)

Splenocytes were stimulated with intact virions at 37 °C for three days. Supernatants were harvested and subjected to cytometric bead array (CBA, BD Biosciences) analysis to detect levels of Th1/Th2/Th17 cytokines, tumor necrosis factor alpha (TNF-α), IFN-γ, interleukin 2 (IL-2), IL-4, IL-6 IL-10, IL-17A.

### Statistical analysis

All results are expressed as the means ± standard errors of the means and compared by one-way ANOVA. Statistical analysis was performed using GraphPad Prism™ software (GraphPad, San Diego, CA, USA), and statistical significance was defined as a p value (*p < 0.05, **p < 0.01, ***p < 0.001).

## Results

### MAV/06 vaccine elicits cross-reactive antibodies to multiple wild-type VZV isolates

**Fig. 1:**
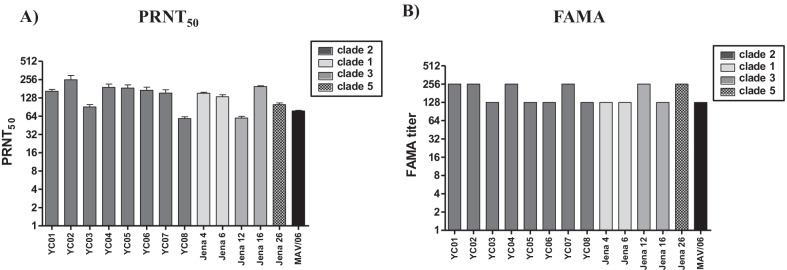
Evaluation of cross reactivity of antibodies induced by MAV/06 vaccine. Guinea pig (n = 30) were immunized subcutaneously two times at three weeks’ interval with MAV/06 vaccine. Sera were collected by cardiac puncture 3 weeks after second immunization. **A** PRNT_50_ titer against multiple VZV isolates (included genetic clade 1–5) were measured by plaque reduction neutralization assay in triplicate. Data are presented as means and standard deviations. **B** FAMA, which is the gold standard method to detect protective antibody against VZV, were performed once with MRC-5 cell infected with multiple VZV isolates

Guinea pigs were immunized on Day 0 and 21 subcutaneously in the scruff of the neck with new MAV/06 vaccine. On Day 42 post-immunization, blood was collected and sera were evaluated for capacity to neutralize the infectivity of virion and infected cells with multiple wild-type VZV isolates. Eight YC isolates, isolated in Korea, were projected into clade 2 genotype [27] and six Jena isolates were clustered into major VZV genotype 1, 3 and 5 respectively [21] (Table 1). The neutralization titer against MAV/06 virus was 78 ± 1. The titers against clade 2 viruses, which MAV/06 belongs to, was 159 ± 61 and those for other clades including clade 1, 3, 5 was 129 ± 52 (Fig. 1A, Additional file 1: Table S1). FAMA test, which is to detect antibodies against virus infected cells, is the gold standard method to measure protective antibodies against VZV [30, 31] since VZV is transmitted by cell-to-cell spread. To demonstrate the immunogenicity, PRNT_50_ test was conducted in triplicate and FAMA assay was additionally conducted once as a supplementary assay. As shown in Fig. 1B, FAMA titer was 128 for MAV/06 itself and the titer showed 192 ± 68 for clade 2 viruses and 179 ± 70 for other clades, respectively (Fig. 1B, Additional file 1: Table S2). The neutralization activities against virion and infected cells did not show significant difference between clade 2 and other virus clades. In other words, Attenuated MAV/06 induce protecting antibody response to wild-type viral strains of the main VZV clades 1, 3 and 5 as well as clade 2. Therefore, these data demonstrate that MAV/06 vaccine immunization induces broad cross-clade antibody responses.

### MAV/06 vaccine induced humoral immune responses comparable to commercialized VZV vaccine

C57BL/6 mice were immunized subcutaneously with MAV/06 vaccine or commercialized vaccines two times with three weeks’ interval. Blood was gathered 2 weeks after immunization and sera were tested to detect VZV specific antibodies. Despite the value and broad use of FAMA as a “gold-standard” assay, the VZV gpELISA is an acceptable alternative to detect VZV gp-specific antibodies as a result of the strict parameters of the FAMA test [31]. Since the titer of varicella and zoster vaccine is claimed as “minimum” dose, it is impossible to directly compare the new MAV/06 vaccine with commercialized Oka strain and MAV/06 vaccine.

**Fig. 2:**
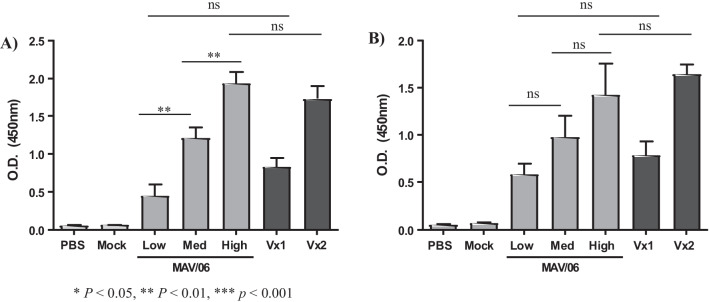
VZV specific antibody responses after MAV/06 vaccine immunization. Mice were immunized intramuscularly two times at three weeks’ interval. MAV/06 vaccines were prepared with different concentration, and commercialized vaccines for chickenpox and shingles were used as positive control. Mock lysate and PBS were used as negative control. **A** Sera were collected 3 weeks after first immunization and **B** 2 weeks after second immunization, and VZV specific antibody were measured with gpELISA. [Vx1: Suduvax, Vx2: Zostavax]. Data are representative of two independent experiments, and presented as means and standard error of the means. ** < 0.01 (ordinary one-way ANOVA). n = 4 mice per group

Regardless, the gpELISA titer of VZV gp-specific antibodies induced by the low dose of new MAV/06 Vaccine (equals to minimum dose of new vaccine) was similar to commercialized Varicella vaccine (Vx1), and the high dose of MAV/06 Vaccine was similar to that of commercialized Zoster vaccine (Vx2) both in priming status (Fig. 2A) and boosting status (Fig. 2B). Antibodies induced by the new MAV/06 vaccine demonstrated a dose-dependent response in priming (Fig. 2A) while the difference was not significant (ns) in boosting status (Fig. 2B).

### MAV/06 vaccine potently increased VZV-specific T cell response

Vaccines containing live organisms are likely to induce cellular immune response as well as humoral immune response [32]. Moreover, T cell mediated immunity in VZV infection had an impact on prognosis for chickenpox and shingles [33]. For these reasons, VZV-specific T cell response was evaluated in new MAV/06 vaccine immunized mice and compared with commercial live vaccine immunized mice.

**Fig. 3:**
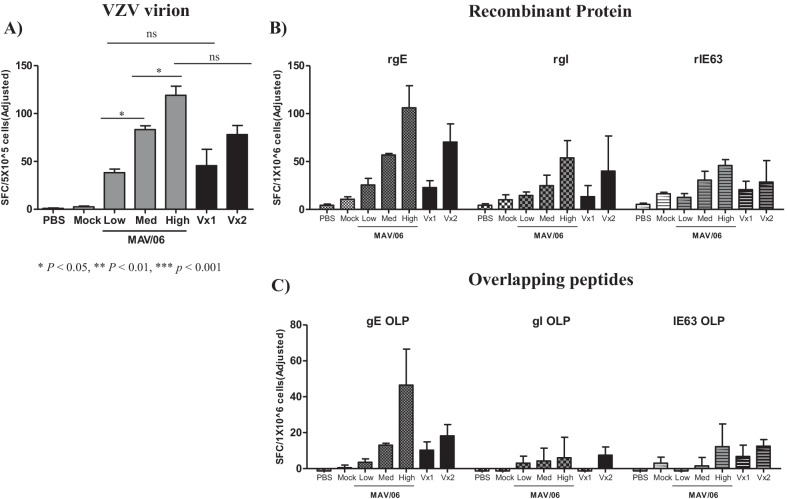
VZV specific T cell responses after MAV/06 vaccine immunization. Splenocyte were collected from immunized mice with MAV/06 or commercialized vaccines. (**A**; VZV lysate) Cells were pulsed with VZV virion or (**B**) recombinant proteins or (**C**; gE, gI and IE63) overlapping peptides as its component. Interferon-γ secreting T cells were measured by IFN-γ ELISPOT. Spot counts were adjusted by baseline spot with mock-pulsed (mock lysate for VZV lysate, medium for recombinant protein, and DMSO for OLP). [Vx1: Suduvax, Vx2: Zostavax]. Data are presented as means and standard error of the means. * < 0.05 (ordinary one-way ANOVA). n = 4 mice per group

Splenocytes from immunized mice were stimulated with VZV virions and IFN-γ secreting T cells were detected in a MAV/06 dose-dependent manner (Fig. 3A). Protein and overlapping peptides for gE, gI and IE63, which is identified as T cell epitopes, were prepared and incubated with splenocytes from vaccine inoculated mice. As shown in Fig. 3B and C, those stimulated cells which is secreting effector cytokine IFN-γ, to a greater or lesser extent compared with commercialized vaccines, and responded better to gE epitope compared with other epitope. This indicated that MAV/06 vaccine is a potent inducer of VZV-specific T cell response.

### MAV/06 vaccine induced Th1 skewed immune response

**Fig. 4:**
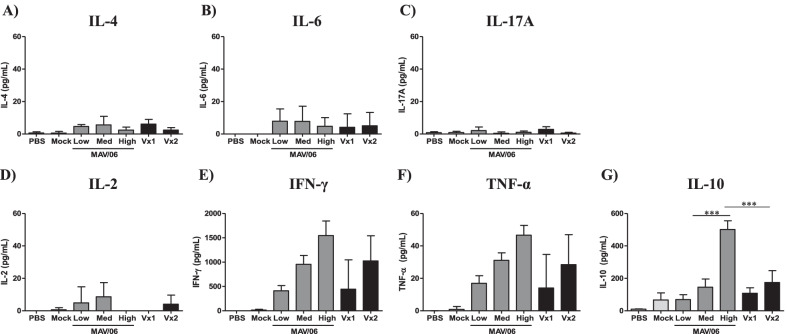
Multiple cytokine analysis of VZV-specific immune cells. Spleen cells were prepared from immunized mice with MAV/06 or commercialized vaccines. Cells were incubated with VZV lysate for 3 days, and supernatants were analyzed with Th1/Th2/Th17 cytokine bead array. Cytokine profiles were adjusted by subtracting result of mock lysate stimulated cell. Data are presented as means and standard deviations. *** < 0.005 (ordinary one-way ANOVA). n = 4 mice per group

Three subsets of CD4+ T helper cells (Th1, Th2 and Th17) have been identified on the basis of cytokine profiles [34, 35]. Th1 cytokines are IL-2, IFN-γ, and TNF-α, and those cytokines promote proliferation, differentiation and activation of macrophage, NK cells, Th1 cells and cytotoxic T cells. Th2 cytokines are IL-4 and IL-6 which stimulates B cell proliferation and maturation into plasma cells. Th17 cytokine is IL17A, and Th17 contributes progression of autoimmune and inflammatory diseases. Splenocyte from immunized mice were incubated with each VZV virion, cytokine profiles in supernatant were analyzed with CBA. IFN-γ, and TNF-α cytokine secreted significantly and the secretion was to a greater or lesser extent comparable in MAV/06 vaccine and commercialized vaccines (Fig. 4E-F) while IL-4, IL-6, IL-17A, IL-2 cytokine was not detected by MAV/06 or commercialized vaccine stimulation (Fig. 4A- 4D). The cells also secreted IL-10 as auto-regulator of Th1 cell activation (Fig. 4G). Taken together, these results indicate that new MAV/06 vaccine induced a Th1 type polarized response comparable to commercialized vaccines.

## Discussion

In this study, we confirmed that the newly developed MAV/06 vaccine triggers humoral immunity by production of antibodies exhibiting cross-reactivity to various VZV virus clade antigens from clade 1, 2, 3, 5. Furthermore, new MAV/06 vaccine induces cell-mediated immunity through Th1 cell response.

In general, vaccines are not administered in different titer as per body weight. The injection volume is the same not only for the adults but also for the infants. Also, for the animal models bigger than rodents, generally the same volume (human dose, 0.5 mL) is administered. However, in the mice model, the antibody titer could be saturated in human dose. For this reason, the injection volume was determined as 5 times lower than the human dose in all mice studies. In addition, the viral titer(PFU/0.5 mL) could be decreased in a time-dependent manner, since VZV vaccines are live-attenuated vaccine. Therefore, the immune responses in various viral titers were evaluated to address the broad range of human dose that could be administered to the subjects.

Currently, there are 7 different clades of VZV worldwide and most of the varicella vaccine viruses come from 5 types of clades [20, 21]. Commercially available VZV vaccines are manufactured from Oka strain and MAV/06 strain from clade 2. Although it is common that confirming reactivity of antibodies induced by vaccination and antigens which is same as the vaccine when conducting PRNT_50_ or FAMA tests, few studies have shown cross-reactivity of vaccine-induced antibodies among different virus clades. Cross-reactivity of antibodies induced by vaccination against various clades of VZV is important to estimate the prophylactic effectiveness against wild-type VZV in the field.

From the PRNT_50_ and FAMA studies, we demonstrated that the newly developed MAV/06 vaccine triggers humoral immunity by the production of antibodies exhibiting cross-reactivity to viruses from VZV clades 1, 2, 3, and 5. Even though the FAMA assay was conducted once, but the reproducibility was identified by the PRNT_50_ results, which was repeated in triplicate. A study conducted with Oka strain illustrated that Oka vaccine induced antibody response to a wild-type VZV that prevailed in Germany [36]. The extent of antibody reactivity to clade 1, 2, 3, and 5 viruses was similar to that of the MAV/06 virus. These results are similar to the results from the study conducted in Germany (Fig. 1) [36]. This study was conducted with the MAV/06 strain that used both in Suduvax and a newly developed Varicella vaccine (Trade name: BARYCELA inj. Reference; https://nedrug.mfds.go.kr/pbp/CCBBB01/getItemDetail?itemSeq=202001448) with upgrade formulation by GC Biopharma Corp. It is the first investigation demonstrating that MAV/06 vaccination induced antibody responses against various VZV clades worldwide as well as clade 2 viruses that prevail in Asian countries, including the Republic of Korea and Japan.

Live-attenuated vaccines exhibit immunological strength that enables antibody induction and other immune reaction such as T cell immune response [32]. Previous studies of VZV vaccines implied the importance of T cell immunity after vaccination [1, 2, 9, 11–14, 33]. The occurrence of herpes zoster and the observed concomitant reduction of cell-mediated immunity (CMI) in individuals with high titers of VZV antibody indicate that the reduction in CMI is the primary cause of herpes zoster. CMI evaluation were performed using the VZV vaccine developed from the Oka strain and reported CMI induction by the VZV vaccine [33, 37]. We investigated MAV/06 vaccine-induced CMI and humoral responses in mice.

Test results of CBA assay showed high amount of secreted IL-10 in the high viral titers compared to commercialized high-dose vaccine, Vx2. IL-10 is known as a cytokine with multiple, pleiotropic, effects in immune-regulation and inflammation. In addition, it also enhances B cell survival, proliferation, and antibody production. For this reason, highly secreted IL-10 level could induce B cell immune responses. However, the appropriate vaccine titers have to be determined by considering various factors such as antibody response, cell-mediated immunity and safety via clinical trials.

We demonstrated the vaccine evoked VZV-specific B cell and T cell immune responses that were comparable to that of the commercial vaccines in mice model. In addition, the new MAV/06 vaccine-induced T cell response was found to be mediated by Th1 rather than Th2 or Th17 cell responses, implying the MAV/06 vaccine functions via intracellular virus clearance as have been shown for other previous studies [38]. These results indicate that the live-attenuated MAV/06 vaccine induces both humoral and cellular immunity in live organism.

Our study provided the explanation to the previously reported vaccine efficacy via immunological characterization of MAV/06 strain and will contribute to future studies for vaccine efficacy or effectiveness after new MAV/06 vaccine.

## Conclusions

This study indicated that MAV/06 strain vaccination triggers B cell immune response to various VZV clades and T cell immune response via Th1 cells in animal model.

## Supplementary Information

**Additional file 1**. **Table S1.** Result of PRNT_50_ titer against multiple VZV isolates. The individual titers against various VZV strains in triplicate including mean and standard deviation values. **Table S2.** Result of FAMA titer against multiple VZV isolates. The individual titers against various VZV strains in FAMA assay.

## Data Availability

The datasets used and/or analyzed during the current study are available from the corresponding author on reasonable request.
