# Supplementary material for: Immunological characteristics of MAV/06 strain of varicella-zoster virus vaccine in an animal model
Source: BMC Immunol. 2022 Jun 3;23:27. doi: 10.1186/s12865-022-00503-6 (PMC9166591; doi:10.1186/s12865-022-00503-6)
Supplement: Supplementary file 1 — Additional file 1. Table S1. Result of PRNT50 titer against multiple VZV isolates. The individual titers against various VZV strains in triplicate including mean and standard deviation values. Table S2. Result of FAMA titer against multiple VZV isolates. The individual titers against various VZV strains in FAMA assay. [file 12865_2022_503_MOESM1_ESM.docx]

**[Supplementary](javascript:;)**[**tables**](javascript:;)

**Table S1. Result of PRNT_50_ titer against multiple VZV isolates.** The individual titers against various VZV strains in triplicate including mean and standard deviation values.

| **Strain name** | **PRNT_50_** | | | **Mean** | **SD** |
| --- | --- | --- | --- | --- | --- |
| YC01 | 156 | 159 | 179 | 165 | 13 |
| YC02 | 304 | 244 | 217 | 255 | 45 |
| YC03 | 99 | 91 | 82 | 91 | 8 |
| YC04 | 162 | 209 | 202 | 191 | 25 |
| YC05 | 197 | 158 | 203 | 186 | 24 |
| YC06 | 178 | 147 | 189 | 172 | 22 |
| YC07 | 149 | 136 | 177 | 154 | 21 |
| YC08 | 57 | 55 | 63 | 58 | 4 |
| Jena 4 | 160 | 148 | 149 | 153 | 6 |
| Jena 6 | 128 | 146 | 128 | 134 | 11 |
| Jena 12 | 64 | 58 | 57 | 60 | 4 |
| Jena 16 | 196 | 192 | 203 | 197 | 6 |
| Jena 26 | 101 | 92 | 105 | 99 | 7 |
| MAV/06 | 78 | 77 | 80 | 78 | 1 |

**Table S2. Result of FAMA titer against multiple VZV isolates.** The individual titers against various VZV strains in FAMA assay.

| **Strain name** | **Clade** | **Titer** |
| --- | --- | --- |
| YC01 | 2 | 256 |
| YC02 | 2 | 256 |
| YC03 | 2 | 128 |
| YC04 | 2 | 256 |
| YC05 | 2 | 128 |
| YC06 | 2 | 128 |
| YC07 | 2 | 256 |
| YC08 | 2 | 128 |
| Jena 4 | 1 | 128 |
| Jena 6 | 1 | 128 |
| Jena 12 | 3 | 256 |
| Jena 16 | 3 | 128 |
| Jena 26 | 5 | 256 |
| MAV/06 | 2 | 128 |
